# Clinical predictors of cybersickness in virtual reality (VR) among highly stressed people

**DOI:** 10.1038/s41598-021-91573-w

**Published:** 2021-06-09

**Authors:** Hyewon Kim, Dong Jun Kim, Won Ho Chung, Kyung-Ah Park, James D. K. Kim, Dowan Kim, Kiwon Kim, Hong Jin Jeon

**Affiliations:** 1 Department of Psychiatry, Hanyang University Medical Center, Seoul, South Korea; 2 Department of Psychiatry, Depression Center, Samsung Medical Center, Sungkyunkwan University School of Medicine, #81 Irwon-ro, Gangnam-gu, Seoul, 06351 South Korea; 3 Department of Health Sciences & Technology, Department of Medical Device Management & Research, and Department of Clinical Research Design & Evaluation, Samsung Advanced Institute for Health Sciences & Technology (SAIHST), Sungkyunkwan University, Seoul, South Korea; 4 Department of Otorhinolaryngology, Samsung Medical Center, Sungkyunkwan University School of Medicine, Seoul, South Korea; 5 Department of Ophthalmology, Samsung Medical Center, Sungkyunkwan University School of Medicine, Seoul, South Korea; 6 AR Lab, Samsung Research, Samsung Electronics Co., Ltd., Seoul, South Korea; 7 Advanced Solution Team, Samsung Research, Samsung Electronics Co., Ltd., Seoul, South Korea; 8 Department of Psychiatry, Kangdong Sacred Heart Hospital, Hallym University College of Medicine, Seoul, South Korea

**Keywords:** Health care, Medical research, Risk factors, Nanoscience and technology

## Abstract

The use of virtual reality (VR) in the treatment of psychiatric disorders is increasing, and cybersickness has emerged as an important obstacle to overcome. However, the clinical factors affecting cybersickness are still not well understood. In this study, we investigated clinical predictors and adaptation effect of cybersickness during VR application in highly stressed people. Eighty-three healthy adult participants with high stress level were recruited. At baseline, we conducted psychiatric, ophthalmologic, and otologic evaluations and extracted physiological parameters. We divided the participants into two groups according to the order of exposure to VR videos with different degrees of shaking and repetitively administered the Simulator Sickness Questionnaire (SSQ) and the Fast Motion sickness Scale (FMS). There was no significant difference in changes in the SSQ or the FMS between groups. The 40–59 years age group showed a greater increase in FMS compared to the 19–39 years age group. Smoking was negatively associated with cybersickness, and a high Positive Affect and Negative Affect Schedule score was positively associated with cybersickness. In conclusion, changing the intensity of shaking in VR did not affect cybersickness. While smoking was a protective factor, more expression of affect was a risk factor for cybersickness.

## Introduction

With the advancement of technology, the use of virtual reality (VR) in the medical field is gradually increasing. VR can be used for various purposes including medical training, surgical planning, education for patients, and rehabilitation^1–3^. In psychiatry, many studies have attempted to deliver exposure therapy by VR and have shown that treatment can be delivered in more accessible and cost-effective manners, compared to conventional exposure therapy^4–9^. Also, research has demonstrated that the efficacy of VR for the reduction of stress or anxiety in the general population is increasing^10–13^.

While the role of VR has broadened in medical fields, cybersickness has emerged as an important obstacle to overcome. Cybersickness is a constellation of symptoms, similar to those of motion sickness, that occur during and after exposure to VR immersion, including eye strain, headache, pallor, sweating, dryness of mouth, fullness of stomach, disorientation, vertigo, ataxia, nausea, and vomiting^14^. In previous studies, about 22–80% of participants have experienced cybersickness during or after application of VR^15–17^. Not only do VR users experience discomfort due to cybersickness, but when VR is used therapeutically, cybersickness can lower compliance with treatment.

Like motion sickness, cybersickness is presumed to occur due to visual-vestibular conflicts, where visual signals give information of bodily movement, but there is no actual movement during VR immersion. This sensory conflict does not match to the individual internal model of central nervous system and this may lead to the symptoms of cybersickness. While the mechanism of cybersickness is still not completely understood, the occurrence of cybersickness is known to largely be determined by individual, device, and task factors^18^. According to previous studies, individual factors include age, sex, illness, and postural instability, as well as chronic insomnia, the tendency to catastrophize somatic symptoms, and activities of the central and autonomic nervous system^14, 16, 19–23^. Device factors include lag, flicker, calibration, and ergonomics^18^. The level of control the user has and duration of VR task are the task factors that affect the occurrence of cybersickness^18^. For example, subjects who have good control in VR are less susceptible to cybersickness, while those with poor control over the VR are more susceptible to cybersickness^24^. And, longer exposure to VR is known to increase the occurrence of cybersickness and severity of symptoms^18^. The degree of immersion to VR is known to have a negative relationship with cybersickness^25^. In addition, when exposed to a task that rotates in VR, cybersickness increases as the rotation speed increases^26^.

Although there are differences between studies, there seems to be an adaptation effect to cybersickness^27^. Although longer exposure to VR is a risk factor for occurrence of cybersickness, previous studies have demonstrated the adaptation effect as the exposure time to VR increases. A study showed that the symptoms of cybersickness worsen as the exposure time increases, but there is a threshold level or time point at which the exacerbation of symptoms stops or decreases, suggesting that the adaptation effect appears in people having long exposure times^27^. Another study found that repetitive exposure to provocative VR content results in habituation^28^. However, still there is insufficient evidence on adaptation effect due to stepwise application of virtual motion that does not match to actual locomotion during VR immersion.

The objective of this study was to investigate clinical predictors and adaptation effect of cybersickness during VR application among highly stressed people. In this study, we hypothesized that: (1) escalating the degree of shaking in VR videos would prevent cybersickness and (2) the clinical variables obtained from psychiatric, ophthalmologic, and otologic evaluations and physiological parameters would predict the occurrence of cybersickness.

## Methods

### Participants

We recruited 83 healthy adult volunteers at least 19 years of age with high stress level from October 2016 to January 2018. We defined high stress as a score of 20 or more on the Perceived Stress Scale-10 (PSS-10)^29^. Given that the occurrence of cybersickness is affected by individual factors and VR has been often used and researched for the reduction of anxiety or stress in psychiatry, we selected the target population as highly stressed but healthy adults in this study. Inclusion criteria were healthy persons who voluntarily participated in this study and had no problems understanding study procedures and controlling VR equipment. Those who had major depressive disorder, bipolar disorder, schizophrenia, other psychotic disorders, delusional disorder, anxiety disorders, delirium, dementia, eating disorders, alcohol use disorder, organic mental disorders, mental retardation, psychiatric disorders due to medical conditions, or suicidal risk were excluded from the study. In addition, those who had neurological illnesses such as stroke or epilepsy and serious medical illnesses were excluded. Those who had medical or surgical history of psychiatric, otologic, or ophthalmologic disorders or problems with neck movements were also excluded. All participants were drug-naive when sample measurement was conducted at the baseline evaluation. At the baseline screening visit, participants were evaluated independently by the psychiatrist (HJ Jeon), the otorhinolaryngologist (WH Chung), and the ophthalmologist (K Park). In order to evaluate psychiatric disorders, a psychologist who specialized in psychiatric evaluation administered the Korean version of the Mini International Neuropsychiatric Interview (MINI)^30^ to the subjects, according to the Diagnostic and Statistical Manual of Mental Disorders (DSM-5)^31^. The study was approved by the Institutional Review Board of the Samsung Medical Center, all experiments were performed in accordance with Good Clinical Practice guidelines, and all participants gave written informed consent at enrollment into the study.

### Baseline evaluation

#### Psychological scales

Participants underwent several psychological scales, including the State-Trait Anxiety Inventory-X1,X2 (STAI-X1,X2)^32^, 0–100 numeric rating scale (NRS)^33^, Positive Affect and Negative Affect Schedule (PANAS)^34^, Sheehan’s Disability Scale (SDS)^35^, and the five-level version of the EQ-5D (EQ-5D-5L)^36^.

#### Physiological parameters

Using a computerized biofeedback system, ProComp Infiniti^37^ (Thought Technology Ltd., Montreal, Canada), physiological parameters were obtained through sensors attached to each subject’s body. These physiological parameters included electromyography (EMG), skin conductance, skin temperature, respiration amplitude, and heart rate/blood vessel pressure (HR/BVP), along with heart rate variability (HRV) parameters such as the HR from the inter-beat interval (IBI), very low frequency band (VLF), low frequency band (LF), high frequency band (HF), LF/HF ratio, number of interval differences of successive normal-to-normal (NN) intervals greater than 50 ms (NN50), standard deviation of NN (SDNN), and the root mean square of the successive differences (RMSSD). Parameters extracted through monitoring for 3 min and 30 s at baseline were included in the analysis. EMG was recorded through the surface EMG sensors those were placed on the skin’s surface. The sensors can record EMG signals of up to 1600 microvolts (µV), root mean square and measure muscle activity. Skin conductance was measured through two electrodes those were strapped to two fingers of one hand. Skin conductance represents changes in the sympathetic nervous system. When a subject becomes more or less stressed, the skin conductance increases or decreases proportionally. Skin temperature was recorded through thermistor, which were strapped to the dorsal or palmar side of a finger. The peripheral temperature varies according to the amount of blood perfusing the skin and dependent on a subject’s state of sympathetic arousal. As a subject gets stressed, their extremities tend to get colder. Respiration amplitude was recorded through the respiration sensor that were strapped around a subject’s abdomen. It detects the expansion and contraction of the rib cage or abdominal area and converts it as a graph on the screen. The respiration amplitude is a relative measure of chest expansion and does not have standard units.

#### Ophthalmologic parameters

In the ophthalmologic evaluation, subjective visual fatigue scale, tear breakup time (TBUT), pupillometry, near point of accommodation (NPA), near point of convergence (NPC), and inter-blink interval were measured.

#### Otologic parameters

In the otologic evaluation, the video head impulse test (VHIT) and sensory organization test (SOT) were conducted.

### Application of VR

**Figure 1:**
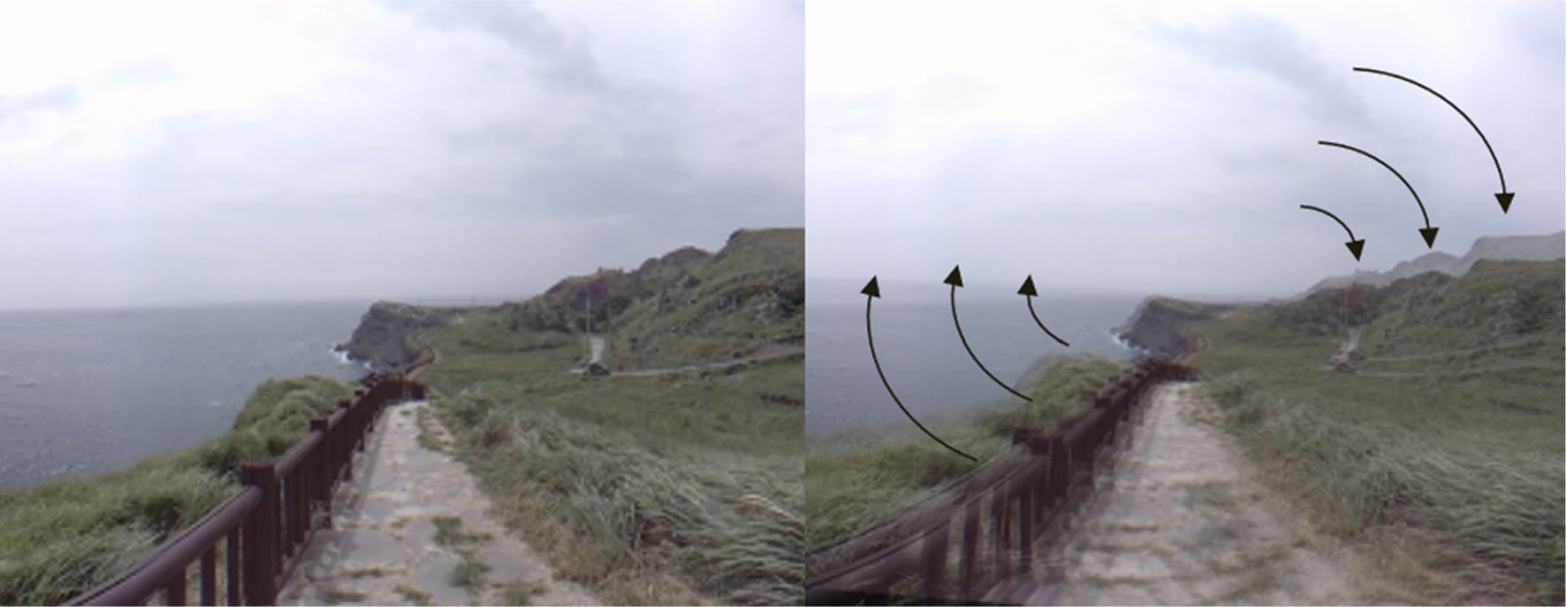
The shaking and dizzy virtual reality (VR) video. The original video (left) was artificially modified for this study by adding a roll swing of the sine waveform at 30 Hz in the Z-axis direction with 0.008°/s for each grade. Then image movements of 0.3°/s (VR with less shaking) and 0.38°/s (VR with more shaking) were made.

Participants were exposed to shaking and dizzy immersive VR videos while sitting on a chair. The original video was provided by Korea Land and Geospatial Informatix Corporation and artificially modified for this study by adding a roll swing of the sine waveform at 30 Hz in the Z-axis direction with 0.008°/s for each grade (Fig. 1). Then we made image movements of 0.3°/s (VR with less shaking) and 0.38°/s (VR with more shaking). Participants were exposed to a VR video that involved walking on a shaky path for 3 min and 30 s, and after a break of 3 min and 30 s, they were exposed to another VR video that differed in the intensity of shaking from the first video. Among the total study group, VR was applied in the order of escalating degrees of shaking for 40 people and in the order of de-escalating degrees of shaking for the other 43 people (Supplementary Fig. 1). During the exposure to the VR videos, participants were asked to count the number of persons who appeared in the video in order to increase their attention to it. The study was conducted in a room that was exclusively prepared to block outside noise in the Clinical Trial Center located in Samsung Medical Center. Samsung Gear VR (Samsung Electronics Co., Ltd., Suwon, South Korea) was used for the study, and the head-mounted display (HMD) device included separate screens for each eye, integrated head-tracking, and stereo earphones.

### Outcomes

To measure cybersickness, the Simulator Sickness Questionnaire (SSQ)^38^ and the Fast Motion Sickness Scale (FMS)^39^ were used. The SSQ and FMS were administered before VR application and again immediately after application of each VR video. The primary outcomes were the changes in SSQ and FMS scores.

### Statistical analyses

We examined the distribution of demographic characteristics, baseline psychological scales, physiological parameters, ophthalmologic parameters, and otologic parameters. Categorical variables were presented as frequencies with percentages and continuous variables as means with standard deviations (SD). The changes in the SSQ and FMS according to the order of exposure to VR videos, age, and sex were analyzed using either a Student’s t-test or an analysis of variance (ANOVA). To confirm the correlation between potential factors and the changes in SSQ or FMS, Pearson’s correlation or Spearman’s rank correlation was used depending on the characteristics of variables. Variables with a p-value below 0.10 were included in multivariable regression analyses. We reported *β*-coefficients and p-values. We considered a p-value of less than 0.05 as statistically significant. All statistical analyses were performed using IBM SPSS Statistics Software (version 24; IBM, New York, USA).

## Results

### Baseline characteristics of participants

Table 1 shows the demographic characteristics and baseline clinical evaluation data of the participants. Among 83 total participants, 48.2% were male and 51.8% were female. The mean age of the participants was 38.53 years old. The mean baseline SSQ was 23.76 (SD = 26.29). The responses of subjects to baseline SSQ are presented in Supplementary Table 1.

**Table 1:** Baseline characteristics of total participants.

	N = 83	
	Number	%
**Sex**		
Male	40	48.2
Female	43	51.8
**Alcohol drinking**		
Yes	49	59.0
No	34	41.0
**Smoking**		
Yes	17	20.5
No	66	79.5
**Motion sickness**		
Yes	18	21.7
No	65	78.3

	**Mean**	**SD**
Age	38.53	11.80
Baseline SSQ	23.76	26.29
Nausea	16.09	21.89
Oculomotor	20.82	21.60
Disorientation	27.67	36.08
Total	23.76	26.29
**Psychological scales**		
STAI-X-1	46.95	10.01
NRS	55.52	24.17
PSS-10	25.93	4.50
PANAS—total	22.55	9.80
PANAS—positive affect	13.89	6.32
PANAS—negative affect	8.69	6.64
SDS	15.08	6.73
STAI-X-2	47.82	10.00
EQ-5D-5L	6.47	1.63
**Physiological parameters**		
EMG (µV)	2.27	1.33
Skin conductance (µS)	0.56	0.59
Skin temperature (℃)	33.03	1.48
Respiration amplitude	42.15	6.87
HR/BVP	29.70	0.09
HR from IBI	70.54	11.94
VLF total	61.95	47.84
LF total	93.94	89.80
HF total	79.55	84.29
HRV total	247.82	201.18
LF/HF	2.15	2.20
EKG IBI	848.96	154.97
NN50	62.40	72.16
SDNN	59.00	62.14
RMSSD	76.81	104.49
**Ophthalmologic parameters**		
Subjective visual fatigue	4.06	3.78
TBUT	4.97	2.64
Maximal pupil diameter	4.72	1.02
Minimal pupil diameter	2.54	0.93
Pupil constriction percentage	− 43.36	20.81
Latency of papillary response	0.22	0.05
Near point of accommodation	7.84	4.51
Near point of convergence	7.45	5.12
Inter-blink interval	29.83	16.29
**Otologic parameters**		
VHIT	1.05	0.07
SOT (equilibrium)	81.30	5.30
SOT (somatosensory)	0.98	0.02
SOT (visual)	0.88	0.06
SOT (vestibular)	0.72	0.11
SOT (preference)	1.04	0.05

*SD* standard deviation, *SSQ* Simulator Sickness Questionnaire, *STAI* State-Trait Anxiety Inventory, *NRS* Numeric Rating Scale, *PSS-10* Perceived Stress Scale, *PANAS* Positive and Negative Affect Schedule, *SDS* Sheehan Disability Scale, *EQ-5D-5L* Five-level version of EQ-5D, *EMG* electromyography, *HR/BVP* heart rate/blood vessel pressure, *IBI* inter-beat interval, *VLF* very low frequency band, *LF* low frequency band, *HF* high frequency band, *HRV* heart rate variability, *NN50* number of interval differences of successive normal-to-normal (NN) intervals greater than 50 ms, *SDNN* standard deviation of NN, *RMSSD* the root mean square of the successive differences, *TBUT* tear breakup time, *VHIT* video head impulse test, *SOT* sensory organization test.

### Change in SSQ and FMS after application of VR

Table 2 shows the change in SSQ and FMS after application of VR according to the order of exposure to VR, age group, and sex. There was no significant difference in the changes in SSQ and FMS between the group exposed to VR videos in order of increasing degrees of shaking and the group exposed to VR videos in order of decreasing degrees of shaking.

**Table 2:** Change in SSQ and FMS after application of VR according to shaking of VR, age group, and sex.

	Order of exposure to VR			Age group 1					Age group 2			Sex		
	Increasing degree of shaking (n = 40)	Decreasing degree of shaking (n = 43)	*P*	19 ~ 29 (n = 21)	30–39 (n = 21)	40–49 (n = 23)	50–59 (n = 18)	*P*	19–39 (n = 42)	40–59 (n = 41)	*P*	Male	Female	*P*
	Mean (SD)			Mean (SD)					Mean (SD)			Mean (SD)		
ΔSSQ	35.15 (45.25)	31.41 (45.73)	0.709	23.43 (41.26)	32.77 (37.97)	38.61 (54.82)	38.23 (45.93)	0.683	28.10 (39.47)	38.44 (50.49)	0.301	29.60 (49.99)	36.57 (40.69)	0.487
ΔFMS	6.07 (5.09)	5.95 (4.62)	0.136	3.87 (3.42)	4.68 (4.80)	7.84 (5.71)	7.65 (3.77)	0.009	4.28 (4.14)	7.76 (4.90)	0.001	4.93 (4.23)	6.98 (5.18)	0.053

*SSQ* Simulator Sickness Questionnaire, *FMS* Fast Motion sickness Scale, *VR* virtual reality, *SD* standard deviation.

According to age group, although there was no difference in the change of SSQ between groups, the changes in FMS in the 19–29, 30–39, 40–49, and 50–59 age groups were 3.87 (SD = 3.42), 4.68 (SD = 4.80), 7.84 (SD = 5.71), and 7.65 (SD = 3.77), respectively, and there was a significant difference between groups with *p*-value of 0.009. When age groups were divided as 19–39 and 40–59, the difference between age groups was more evident, with the changes in FMS being 4.28 (SD = 4.14) and 7.76 (SD = 4.09), respectively (*p*-value = 0.001).

According to sex, the SSQ increased by 29.60 (SD = 49.99) in males and 36.57 (SD = 40.69) in females. The FMS increased by 4.93 (SD = 4.23) in males and 6.98 (SD = 5.18) in females. Both SSQ and FMS increased more in females, but the differences with males were not statistically significant.

### Correlation between factors and cybersickness

We confirmed the correlation between variables and the change of SSQ or FMS. Among all clinical and physiological variables, smoking, NRS, PANAS-total, PANAS-positive affect, PANAS-negative affect, NPA, and NPC showed significant correlations with the change in SSQ, while smoking, age, PANAS-total, and NPC showed significant correlations with the change in FMS (Table 3).

**Table 3:** Correlation analysis.

	SSQ		FMS	
	Spearman’s rho	*P*	Spearman’s rho	*P*
Sex	0.201	0.069	0.207	0.060
Motion sickness	0.138	0.213	0.133	0.232
Alcohol drinking	0.020	0.854	0.055	0.619
Smoking	− 0.283	0.010	− 0.319	0.003
Group	− 0.071	0.526	− 0.010	0.928

	Pearson’s r	*P*	Pearson’s r	*P*
Age	0.170	0.125	0.339	0.002
Baseline SSQ	0.193	0.081	0.201	0.068
**Psychological scales**				
STAI-X-1	0.175	0.114	0.090	0.421
NRS	0.218	0.047	0.091	0.412
PSS-10	− 0.187	0.091	− 0.014	0.901
PANAS—total	0.390	0.000	0.258	0.019
PANAS—positive affect	0.311	0.004	0.196	0.076
PANAS—negative affect	0.284	0.009	0.198	0.073
SDS	0.059	0.596	0.054	0.625
STAI-X-2	0.126	0.256	0.093	0.401
EQ-5D-5L	0.106	0.342	0.110	0.322
**Physiological parameters**				
EMG	− 0.100	0.371	− 0.034	0.760
Skin conductance	0.021	0.851	− 0.085	0.443
Temperature	− 0.006	0.958	− 0.054	0.628
Respiratory	0.060	0.589	0.030	0.785
HR/BVP	0.157	0.156	0.037	0.740
HR from IBI	− 0.001	0.994	− 0.138	0.216
VLF total	− 0.036	0.748	− 0.051	0.649
LF total	− 0.068	0.544	− 0.130	0.249
HF total	0.013	0.911	− 0.041	0.719
HRV total	− 0.031	0.786	− 0.083	0.461
LF/HF	− 0.193	0.085	− 0.182	0.104
EKG IBI	0.216	0.052	0.179	0.108
NN50	0.004	0.974	− 0.047	0.678
SDNN	0.112	0.317	0.045	0.690
RMSSD	0.098	0.381	0.011	0.924
**Ophthalmologic parameters**				
Subjective visual fatigue	0.078	0.481	0.156	0.159
TBUT	− 0.115	0.323	− 0.163	0.160
Maximal pupil diameter	0.159	0.168	0.033	0.773
Minimal pupil diameter	0.020	0.862	− 0.048	0.680
Pupil Constriction percentage	0.029	0.802	0.012	0.918
Latency of papillary response	0.120	0.306	− 0.050	0.667
Near point of accommodation	− 0.240	0.036	− 0.201	0.079
Near point of convergence	− 0.330	0.003	− 0.298	0.009
Inter-blink interval	− 0.215	0.060	− 0.195	0.088
**Otologic parameters**				
VHIT	0.052	0.693	0.015	0.911
SOT (equilibrium)	0.045	0.735	− 0.161	0.218
SOT (somatosensory)	0.024	0.853	− 0.196	0.133
SOT (visual)	0.034	0.799	− 0.092	0.484
SOT (vestibular)	0.037	0.782	− 0.108	0.411
SOT (preference)	0.045	0.733	− 0.002	0.987

*SSQ* Simulator Sickness Questionnaire, *FMS* Fast Motion sickness Scale, *STAI* State-Trait Anxiety Inventory, *NRS* Numeric Rating Scale, *PSS-10* Perceived Stress Scale, *PANAS* Positive and Negative Affect Schedule, *SDS* Sheehan Disability Scale, *EQ-5D-5L* Five-level version of EQ-5D, *EMG* electromyography, *HR/BVP* heart rate/blood vessel pressure, *IBI* inter-beat interval, *VLF* very low frequency band, *LF* low frequency band, *HF* high frequency band, *HRV* heart rate variability, *NN50* number of interval differences of successive normal-to-normal (NN) intervals greater than 50 ms, *SDNN* standard deviation of NN, *RMSSD* the root mean square of the successive differences, *TBUT* tear breakup time, *VHIT* video head impulse test, *SOT* sensory organization test.

### Multivariable linear regression analyses

Multivariate linear regression analyses were performed, including 14 variables identified as having a p-value below 0.10 in the correlation analyses. Regarding PANAS, we performed multivariate regression analyses with two models, one including the PANAS-total variable and another model including the PANAS-positive affect and PANAS-negative affect variables. Table 4 shows the result of the multivariate analysis including PANAS-total. For SSQ, smoking was associated with reduced SSQ (β = − 31.29, *p* = 0.017, 95% CI − 56.68, − 5.91), and PANAS-total was associated with increased SSQ (β = 1.58, *p* = 0.004, 95% CI 0.53, 2.62). Similar to FMS, smoking was associated with reduced FMS (β = − 2.60, *p* = 0.049, 95% CI − 5.18, − 0.02), and PANAS-total was associated with increased FMS (β = 0.12, *p* = 0.033, 95% CI 0.01, 0.22). Supplementary Table 2 shows the result of multivariate analysis including PANAS-positive affect and PANAS-negative affect instead of PANAS-total. For SSQ, smoking was associated with reduced SSQ (β = − 34.47, *p* = 0.009, 95% CI − 59.94, − 9.00), and PANAS-positive affect was associated with increased SSQ (β = 2.63, *p* = 0.004, 95% CI 0.89, 4.36). For FMS, smoking was associated with reduced FMS (β = − 2.74, *p* = 0.041, 95% CI − 5.37, − 0.11).

**Table 4:** Multivariable linear regression analysis.

	SSQ				FMS			
	*β*-coefficient	Standard error	*P*	95% CI	*β*-coefficient	Standard error	*P*	95% CI
Sex	− 3.43	11.69	0.770	− 26.80, 19.95	1.25	1.19	0.297	− 1.12, 3.63
Smoking	− 31.29	12.70	0.017	− 56.68, − 5.91	− 2.60	1.29	0.049	− 5.18, − 0.02
Age	− 0.27	0.50	0.592	− 1.26, 0.72	0.09	0.05	0.074	− 0.01, 0.19
Baseline SSQ	− 0.02	0.19	0.928	− 0.41, 0.37	0.01	0.02	0.601	− 0.03, 0.05
NRS	0.29	0.23	0.210	− 0.17, 0.75	− 0.03	0.02	0.176	− 0.08, 0.01
PSS-10	− 1.67	1.33	0.212	− 4.33, 0.98	0.25	0.14	0.067	− 0.02, 0.52
PANAS—total	1.58	0.52	0.004	0.53, 2.62	0.12	0.05	0.033	0.01, 0.22
LF/HF	− 0.98	2.44	0.691	− 5.86, 3.91	− 0.13	0.25	0.604	− 0.63, 0.37
EKG IBI	0.03	0.03	0.370	− 0.04, 0.09	0.00	0.00	0.390	0.00, 0.01
Near point of accommodation	1.72	1.89	0.366	− 2.06, 5.51	0.12	0.19	0.539	− 0.27, 0.50
Near point of convergence	− 2.67	1.67	0.115	− 6.01, 0.67	− 0.29	0.17	0.093	− 0.63, 0.05
Inter-blink interval	− 0.58	0.32	0.079	− 1.23, 0.07	− 0.05	0.03	0.172	− 0.11, 0.02

Explanatory variables with a p-value below 0.10 in correlation analyses were included in multivariate linear regression analyses.

*SSQ* Simulator Sickness Questionnaire, *FMS* Fast Motion sickness Scale, *CI* confidence interval, *NRS* Numeric Rating Scale, *PSS-10* Perceived Stress Scale, *PANAS* Positive and Negative Affect Schedule, *LF* low frequency band, *HF* high frequency band, *IBI* inter-beat interval.

## Discussion

In this study, we identified the adaptation effect according to varying degrees of shaking of VR videos and clinical predictive factors of cybersickness. When participants were exposed to two VR videos with different degrees of shaking in different orders, there was no difference in cybersickness between groups. There was a difference in the occurrence of cybersickness according to age group, and it was higher in the 40–59 age group compared to the 19–39 age group. Multivariate regression showed smoking was a factor that prevented cybersickness, and a high PANAS score was identified as a risk factor for cybersickness.

In this study, changing the intensity of shaking of VR videos did not affect cybersickness. Although previous studies have demonstrated adaptation effect according to exposure time^27^ or repetition of exposure to VR^28^, this study did not show adaptation effect when the intensity of shaking of VR was increased or decreased. These findings suggest that regarding adaptation or habituation of cybersickness, exposure time to VR affect more sensitively than changing in intensity of sensory stimuli. The effect of the interaction between the intensity of stimulation and exposure time on cybersickness can be investigated in future studies.

In this study, we tried to determine clinical predictors of cybersickness through psychiatric, ophthalmologic, and otological evaluation and extraction of physiological parameters. Among ophthalmological and otological parameters, although NPA and NPC in pupils showed correlations with changes of the SSQ and the FMS, multivariate regression analyses did not show significance after adjusting the other factors. This finding suggests ophthalmological or otological impairment does not aggravate cybersickness. Previous studies have reported that motion sickness occur mostly in people with intact vestibular system^40, 41^. Patients with impaired labyrinthine function do not normally experience classical motion sickness, but partially experience visually-induced motion sickness^40, 41^. Likewise, recent evidence reports that individuals with a greater sensitivity to visual stimuli are more likely to experience more discomfort during VR applications^42^.

Previous studies have shown that smokers tend to have less motion sickness or postoperative nausea and vomiting, while nicotine nasal spray increases sensitivity to motion sickness. As an explanation of this, temporary nicotine withdrawal may lead to increased tolerance for motion sickness or nausea^43, 44^. Nicotine affects motion sickness through the mechanism of the nicotinic cholinergic receptor (nAchR), which regulates the excitability of the caudal vestibular nucleus (CVN)^45^. The CVN contributes to both cardiovascular controls during head movements and autonomic manifestations of motion sickness through its strong connection with brain stem autonomic areas, such as the solitary tract nucleus and the parabrachial nucleus^46–50^. Smoking was a protective factor in our study, and it is thought that the short-term deprivation of nicotine may affect the result by this mechanism.

The high baseline score of PANAS was associated with an increased risk of cybersickness. All three PANAS variables, the total score, positive affect, and negative affect, showed significant correlations with SSQ, and in regression analyses, the total score of PANAS showed a significant association with changes in both SSQ and FMS. Also, the positive affect score of PANAS was associated with a change in SSQ in regression analysis. Because PANAS represents the expression of both positive and negative affect, our results suggest that more expression of affect is associated with cybersickness. Also, although there was no significant association with change in SSQ or FMS, the baseline score of 0–100 NRS, which measures subjective discomfort, was positively correlated with the change in SSQ. In previous studies, although the evidence of an association between cybersickness and affective expression or subjective discomfort is lacking, there has been evidence that emotional distress is associated with cybersickness. A study showed that there was no difference in simulator-related side effects between groups when exposure therapy was performed both conventionally and by VR in PTSD patients, and the authors suggested that it is possible that anxiety rather than VR accounts for any simulator-related side effects^51^. Another study found that anxiety has a mediating effect on cybersickness that occurs during VR application^52^. In addition, anxiety-related personality traits are known to affect visual and vestibular control of balance^53–58^. Another study found that being in a VR does not cause anxiety by itself, but simulated motion can lead to anxiety^59^. There is also an evidence that the neuroticism personality trait is a mediating factor in the relationship between anxiety and the visuo-vestibular system. A study conducted that used a VR rollercoaster task found that neuroticism modulates the brain visuo-vestibular and anxiety systems during VR application^60^. In addition, in patients with persistent postural perceptual dizziness (PPPD), characterized by persistent dizziness and unsteadiness and exacerbated by upright posture, self-motion, and exposure to complex or moving visual stimuli, the neurotic personality trait was associated with brain regions mediating attention to visual motion cues^61^. These studies suggest that an individual’s personality traits and anxiety may be more decisive predictors of cybersickness than the visuo-vestibular system. Given that an emotional state such as anxiety and personality traits such as neuroticism are related to cybersickness and influence each other, physicians should select VR content carefully, especially when using VR for the reduction of anxiety or stress.

This study has several limitations. First, since this study targeted a high stress group, it is difficult to apply the results of this study directly to the general population. However, if VR is used for anxiety or stress reduction, it is likely that users will have high stress. Moreover, considering that cybersickness is related to emotional expression or distress, results in the high stress group may be more applicable in clinical settings. In this study, screening was performed using the PSS-10, but in future studies, research on cybersickness in various target groups is expected to broaden accessibility to VR. Second, we investigated the adaptation effect using two VR videos with different degrees of shaking for 3 min and 30 s each, and there was no adaptation effect observed by this method. However, we should not conclude that there is no adaptation effect in cybersickness during VR application. According to previous studies, the presence of an adaptation effect depends on the methodology. In particular, when the same VR contents were applied, an adaptation effect appeared when the exposure time was prolonged. In the present study, the exposure time to each VR video was 3 min and 30 s, and it is likely that the specific methodology such as duration of exposure or degree of shaking will determine if there is an adaptation effect. Third, we applied VR to the subjects for a total of 7 min, but this time may not be enough to induce cybersickness. A previous systematic review reported that cybersickness appears approximately 10–15 min after VR immersion^62^. Fourth, subjects were applied VR while sitting on a chair without actual locomotion. Therefore, the cybersickness induced in this study may have different mechanism with that occurring in a more interactive experience during VR application.

Despite these limitations, our study has the following strengths. To our knowledge, this is the first study investigating the adaptation effect of cybersickness when applying VR with different orders of exposure to VR videos with different degrees of shaking to participants. Although there was no adaptation effect observed in the results of this study, the application of VR in clinical settings will be further expanded as evidence is accumulated in future research. In addition, we identified clinical risk factors for cybersickness, including a comprehensive assessment of psychiatric, ophthalmological, and otologic assessments, as well as analysis of physiological parameters, which are promising biomarkers for psychiatric diagnosis.

The convergence of medicine and new technology is gradually expanding. In psychiatry, cybersickness is an important issue to overcome for interventions such as exposure therapy in PTSD or anxiety disorders and relaxation in a high stress population to be performed efficiently. Cybersickness can not only cause discomfort during the medical use of VR, but it can also make patients reluctant to use VR, thereby reducing its accessibility. In addition, although not replicated in this study, there is also evidence that cybersickness causes a change in heart rate, cutaneous thermoregulatory vascular tone, and prolongation of reaction time^63^. Another study found a change in brain perfusion during the experience of cybersickness^64^. These results suggest that cybersickness should also be handled in terms of safety, and clinicians should prepare for multiple scenarios before the application of new technologies.

In conclusion, the order of applying VR with different degrees of shaking did not affect to cybersickness. While smoking was a protective factor, more expression of affect was a risk factor for cybersickness.

## Supplementary Information

Supplementary Information.
