# Supplementary material for: Clinical predictors of cybersickness in virtual reality (VR) among highly stressed people
Source: Sci Rep. 2021 Jun 9;11:12139. doi: 10.1038/s41598-021-91573-w (PMC8190110; doi:10.1038/s41598-021-91573-w)
Supplement: Supplementary file 1 — Supplementary Information. [file 41598_2021_91573_MOESM1_ESM.pdf]

Supplementary Figure 1. Study design.

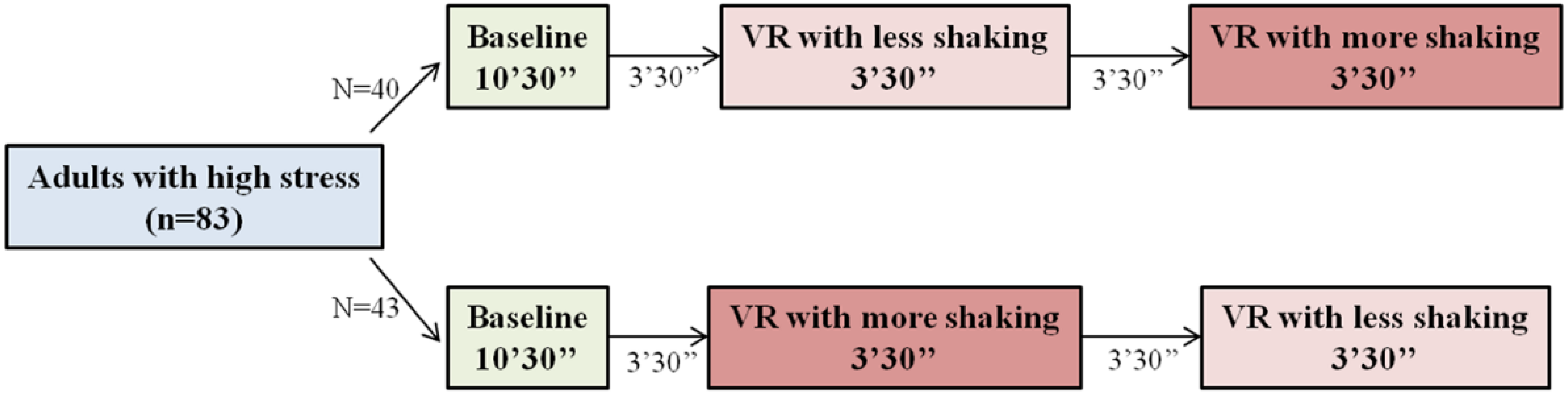

Supplementary Table 1. The responses of subjects to baseline SSQ

	**0 (No symptom)**	**1 (Mild)**	**2 (Moderate)**	**3 (Severe)**
	**N (%)**			
General discomfort	53 (63.9)	25 (30.1)	5 (6.0)	0 (0)
Fatigue	40 (48.2)	35 (42.2)	6 (7.2)	2 (2.4)
Headache	60 (72.3)	21 (25.3)	2 (2.4)	0 (0)
Eyestrain	38 (45.8)	40 (48.2)	5 (6.0)	0 (0)
Difficulty focusing eyes	64 (77.1)	17 (20.5)	2 (2.4)	0 (0)
Increased salivation	71 (85.5)	11 (13.3)	1 (1.2)	0 (0)
Increased sweating	69 (83.1)	11 (13.3)	3 (3.6)	0 (0)
Nausea	63 (75.9)	17 (20.5)	3 (3.6)	0 (0)
Difficulty concentrating	60 (72.3)	21 (25.3)	2 (2.4)	0 (0)
Fullness of head	58 (69.9)	20 (24.1)	5 (6.0)	0 (0)
Blurred vision	64 (77.1)	19 (22.9)	0 (0)	0 (0)
Dizziness with eyes open	59 (71.1)	22 (26.5)	2 (2.4)	0 (0)
Dizziness with eyes closed	64 (77.1)	18 (21.7)	1 (1.2)	0 (0)
Vertigo	61 (73.5)	18 (21.7)	4 (4.8)	0 (0)
Stomach awareness	67 (80.7)	13 (15.7)	3 (3.6)	0 (0)
Burping	76 (91.6)	6 (7.2)	1 (1.2)	0 (0)

SSQ, Simulator Sickness Questionnaire

Supplementary Table 2. Multivariable linear regression analysis.

	SSQ				FMS			
	***β*-coefficient**	**Standard error**	***P***	**95% CI**	***β*-coefficient**	**Standard error**	***P***	**95% CI**
Sex	-5.05	11.61	0.665	-28.26, 18.16	1.17	1.20	0.331	-1.22, 3.57
Smoking	-34.47	12.74	0.009	-59.94, -9.00	-2.74	1.31	0.041	-5.37, -0.11
Age	-0.38	0.50	0.444	-1.37, 0.61	0.09	0.05	0.096	-0.02, 0.19
Baseline SSQ	0.01	0.19	0.941	-0.37, 0.40	0.01	0.02	0.558	-0.03, 0.05
NRS	0.45	0.25	0.079	-0.05, 0.95	-0.03	0.03	0.335	-0.08, 0.03
PSS-10	-1.25	1.34	0.357	-3.94, 1.44	0.27	0.14	0.055	-0.01, 0.55
PANAS – positive affect	2.63	0.87	0.004	0.89, 4.36	0.16	0.09	0.074	-0.02, 0.34
PANAS – negative affect	0.62	0.82	0.456	-1.02, 2.26	0.07	0.08	0.394	-0.10, 0.24
LF/HF	-0.58	2.43	0.813	-5.44, 4.29	-0.11	0.25	0.658	-0.61, 0.39
EKG IBI	0.03	0.03	0.364	-0.03, 0.09	0.00	0.00	0.391	0.00, 0.01
Near point of accommodation	2.24	1.90	0.244	-1.57, 6.04	0.14	0.20	0.473	-0.25, 0.53
Near point of convergence	-3.01	1.67	0.076	-6.35, 0.32	-0.31	0.17	0.081	-0.65, 0.04
Inter-blink interval	-0.60	0.32	0.068	-1.24, 0.05	-0.05	0.03	0.168	-0.11, 0.02

Explanatory variables with a p-value below 0.10 in correlation analyses were included in multivariate linear regression analyses

SSQ, Simulator Sickness Questionnaire; FMS, Fast Motion sickness Scale; CI, confidence interval; NRS, Numeric Rating Scale; PSS-10, Perceived Stress Scale; PANAS, Positive and Negative Affect Schedule; LF, low frequency band; HF, high frequency band; IBI, inter-beat interval.
